# A socioeconomic approach to the profile of microcredit holders from the Hispanic minority in the USA

**DOI:** 10.1186/s40854-022-00422-w

**Published:** 2023-01-10

**Authors:** Salvador Cruz Rambaud, Joaquín López Pascual, Emilio M. Santandreu

**Affiliations:** 1 Departamento de Economía y Empresa, Universidad de Almería, 04120 Almería, Spain; 2 Departamento de Economía de la Empresa, Universidad Rey Juan Carlos, 28032 Vicálvaro, Madrid, Spain; 3 OUR Microlending LLC, Miami, FL 33145 USA

**Keywords:** Microcredit, Customer profile, Socioeconomic factors, Multinomial logit regression, Repayment, Microentrepreneurship, G21, G23, O12

## Abstract

The subject of this study is the microcredit market in the USA, more specifically in Florida. The justification for choosing this specific state is the massive presence of the Hispanic population. This will facilitate a generalization of the obtained results to the microcredit market in Latin American countries. Thus, the objective of this study is to analyze the profile of microcredit holders and their companies from socioeconomic and financial points of view. As our data also consider the degree of repayment of the microloans included in the sample, the clients’ profile is related to the punctuality or default of their corresponding loan repayments using the methodology of multinomial logit regression. The variables used in this study refer to personal information concerning borrowers (gender, age, education level, and marital status), the economic situation of their respective companies (closeness to the lender, number of workers, and revenues), and the characteristics of granted loans (principal, term, and purpose). However, the results of the regression show that only two variables are significant at the 5% significance level: the borrower’s age, which has a positive effect on repayment punctuality, and the loan term, which exhibits a negative effect. The findings of this study have clear implications, as they can help lenders design suitable microloans adjusted to customer profiles. Finally, future research should include other demographics and characteristics of affected companies.

## Introduction

In recent decades, microcredits have become an essential source of funding to promote business ventures for poor people, particularly women and minorities. The relevance of the microcredit topic in the past decade can be found in several publications such as Aitken (2013), Mahmood and Mohd Rosli (2013), and more specifically in Ribeiro et al. (2022), who conducted a scientometric analysis and a systematic literature review to identify the trends in microfinance outcomes from the perspective of more vulnerable people. Indeed, the potential recipients of these studies are policymakers, regulators, and academics to examine the requirements for microfinance and identify the most relevant areas of intervention. Studies on microfinance institutions (MFIs) have typically focused on developing and emerging markets without including the USA. However, an analysis of developed countries, such as the USA, is also important, especially focusing on significant minorities such as the Hispanic. There is undoubtedly an increasing importance of Latin American and Hispanic minorities in the USA in many different fields, especially small and medium enterprises. Despite its importance, there is a lack of research on Latin American microcredit holders in this country, as recently indicated by Santandreu et al. (2020). In addition, recent research (Santandreu and López Pascual 2019) confirms the presence of an important and unattended market in this country that is attractive in size and lacks competition. This is the research gap this study intends to cover.

The main objective of this study is, therefore, to examine the main customer profiles of Hispanic and Latin American microcredit holders and microentrepreneurs in the USA, as well as the main characteristics of their companies that could help in identifying the factors that explain the punctuality of repayment of their respective microloans. Moreover, the study highlights how the importance of distinguishing some of these main factors, specifically those of Hispanic ethnicity, can achieve better results in microcredit policies.

The determinants of microcredit repayment have been studied in the USA, more specifically in four of the oldest microcredit programs located in California (Bhatt and Tang 2002). This research stresses the minority factor because different types of clients have been considered: African-American, Latin American, and Asian. More recently, some scholars (Santandreu et al. 2020) have pointed out that according to managers, the determinants of repayment in microcredits are similar for men and women in the USA. As suggested by Wang and Li (2007), the process of the economic incorporation of ethnic minorities and immigrants depends significantly on institutional capacity and the social, cultural, and political resources of local communities, in which MFIs can play a role.

The microfinance movement in the United States seeks to expand economic opportunities for individuals and foster community economic development by providing small loans and other business services to people who have traditionally been underserved by mainstream financial institutions (see https://www.federalreserve.gov/newsevents/speech/bernanke20071106a.htm). MFIs have therefore played an important role in the entrepreneurial empowerment of Latin Americans, particularly Hispanic minorities. This has undoubtedly made a significant contribution to US economic growth. This contribution derives partially from demographic vitality, as Hispanics are the youngest and largest minority group in America and are on a path toward becoming an increasingly large sector of the US labor force. Grameen America, one of the largest microcredit providers in the United States and the nation’s fastest-growing microfinance organization, has been particularly successful in lending to a specific market of low-income Latin women historically excluded from the financial mainstream (https://www.grameenamerica.org/). However, some scholars point out that these two factors color much of the discussion. First, compared to less developed countries, the structure of the USA economy makes starting small businesses much more difficult (Schreiner and Morduch 2002). Second, there is a great diversity of individuals in the USA who have entrepreneurial desires and talent, but lack access to financing (https://www.aspeninstitute.org/blog-posts/what-we-know-about-microcredit-us/). As the former Federal Reserve Chairman (Ben Bernanke) said, the microfinance movement has grown and adapted considerably during its short history in the United States (see https://www.federalreserve.gov/newsevents/speech/bernanke20071106a.htm). Thus, the microcredit market in the USA is increasing. According to Schreiner and Morduch (2002), the sum total of microcredits granted in the USA is small. In order to have an idea of the current dimension in 2021 of the microcredit market in this country, see Table 1.

**Table 1:** Amounts disbursed by the five main microfinance companies in the USA

Microfinance company	Amount disbursed in loans
Pacific Community Ventures	$25 billion
CDC Small Business Finance Corp.	$20.7 billion
BRAC USA	$2.30 billion
Grameen America Inc.	$1.75 billion
Kiva	$1.60 billion

*Source*: Own elaboration

Lieberman et al. (2012) highlighted some lessons that MFIs in the USA could learn from the successes of these institutions in other markets, recognizing that the operating environment for microfinance in the United States is distinctly different from that in less developed countries.

In 2011, after the Great Recession, Walker (2011) stated: “Today, in the United States, the time is right for microlending to have a big impact on American business,” and some of the specific factors that were mentioned to support this observation were very similar, if not the same, as those factors resulting from the COVID-19: corporate downsizing, unemployment, and income disparity. Since then, microcredits have attracted special attention, as microloans have provided opportunities for self-employment (Rubach et al. 2010; Walker 2011). This is the right time in the USA for microcredits to have a significant impact on business, as economic factors encourage people, many women among them, to see a significant opportunity in business development and self-employment if supported by microcredits (Walker 2011).

These circumstances make the USA microcredit market attractive to specialized and successful MFIs such as Pacific Community Ventures (founded in 1998, provides microloans in California), CDC Small Business Finance Corporation (founded in 1978, operates in Arizona, California, and Nevada), and Kiva (founded in 2005 with headquarters in San Francisco). There are even MFIs coming from other parts of the world, such as the Grameen Foundation (which has operated in the USA since 2008 through Grameen America) and BRAC USA (founded in 1972 in Bangladesh).

Florida was selected for this study because of its high Hispanic population. For this purpose, a wide sample of microloans was analyzed to obtain demographic information on microentrepreneurs (gender, age, education level, and marital status), three economic characteristics of their corresponding business activities (closeness to the microlender, number of workers, and revenues), and three characteristics of granted microloans (loan principal, loan term, and loan purpose). The intention is to ascertain the relationship between the aforementioned ten independent variables and the “punctuality of repayments”. As indicated, the obtained results are expected to serve as a first approach to this topic and will help us to anticipate the behavior of similar microclients in other countries characterized by the lack of reliable information about this topic, thus helping to anticipate the behavior of similar clients in other countries with scarce information on microcredits.

The methodology used in this study is a multinomial logit regression because most independent variables are categorical. Moreover, the dependent variable comprises three categories: repayment on time, delinquency, and default. In essence, after applying this methodology to the available data, the results show the possible behavior of socioeconomic and financial factors and the repayment of microcredits by Hispanic and Latin communities in the USA. More specifically, borrower age positively affects the repayment of microloans granted to businesses in the selected sample (exhibiting a negative coefficient), while loan terms exhibit a positive coefficient and consequently negatively affect repayment punctuality. The two variables are significant at the 5% significance level.

The remainder of this study is organized as follows. “Literature review” section presents a revision of the existing literature on this topic, focusing on potential microclient profiles and the main characteristics of microborrowers and microloans. “Microfinance in the USA: some particularities” section analyzes the situation of the microcredit market in the United States to understand the context of the analysis developed in “Empirical evidence from the USA microcredit market” section, which describes the data and methodology employed in this study. “Discussion” section presents the results of this study. Finally, “Conclusions, recommendations, policy implications and future research” section summarizes and concludes the study.

## Literature review

Microfinance institutions, and consequently, microcredits, as their most common product, are gaining greater importance all over the world. Since its inception, microfinance has focused on Asia and Latin America through a process of continuous expansion (Richardson 2009). In effect, most research on microcredits focuses on developing countries. However, our research aims to characterize the profile and risks associated with microcredit holders in the USA from socioeconomic and financial points of view.

**Fig. 1:**
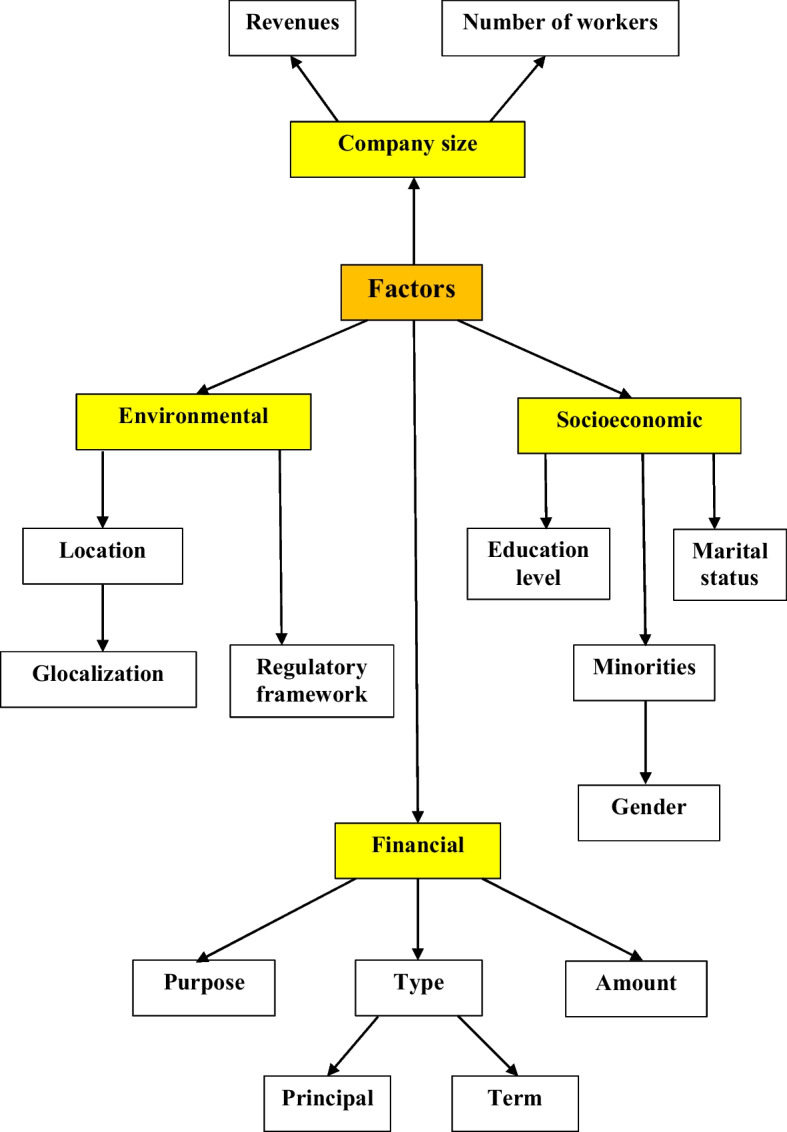
Structure of “Literature review” section. *Source*: Own elaboration

In this section, we analyzed the factors that usually affect microcredit repayment, most of which are socioeconomic in nature (see Fig. 1). Thus, the two approaches in the existing literature can be combined. The first focuses on the main socioeconomic variables involved in granting microloans, and the second focuses on specific socioeconomic factors in the USA. However, other international studies (Deshpande and Burjorjee 2002; Armendariz de Aghion and Morduch 2000, 2005), carried out in several countries and focused on different MFIs without including the USA, consider different factors, mostly socioeconomic, affecting microcredits. In particular, García-Pérez et al. (2017) consider that any research in the field requires an analysis with wide criteria, which should include economic, environmental, social, and governance (EESG) dimensions.

As indicated in Fig. 1, this section is divided into four subsections, which enable better organization of the research results.

### Environmental factors

One of the first factors to be analyzed is the environment. Some schools of thought maintain that a microcredit system suitable for one context may not be appropriate for another. Bhatt and Tang (2001) recommended different microcredit programs for different needs, as differences inevitably exist between communities. In addition, Ayayi and Sene (2010) claimed that adaptation to the environment and clients is necessary, demonstrating that offering microfinance services that are not adapted to clients and their environment hinders MFI viability.

In particular, this study highlights the location (i.e., the specific place where an MFI chooses to place its business) as a relevant factor because the majority of studies on this topic have specifically focused on various non-developed countries (Hulme 1991; Abdullah and Quayes 2016). In effect, the topic of the factors affecting microloan repayment in several different countries has been studied. However, all the microborrowers analyzed in this study are located in the USA (more specifically, in Florida). In this study, the consideration of location was replaced by the distance between the borrower and lender (or, alternatively, the closeness of the borrower to the lender). Analogously, Kassegn and Endris (2022) considered location as the road distance between the smallholder farmer’s residence and the market center in Ethiopia. On the other hand, some scholars considered another factor linked to location, which is glocalization, used to describe a product or service that is developed and distributed globally but is designed for the user or consumer in a local market and adapted to local laws, customs, and preferences. Echoing this glocalization, Santandreu and López Pascual (2019) considered that MFIs, wishing to enter a domestic market (taken to apply to the USA in this study), should “glocalize” or adapt their microcredit policies to some specific groups such as women, as well as their product design and policies for granting microloans.

This study shows that foreign MFIs which decide to enter the US should take appropriate measures to glocalize their microcredit policies, choose microcredit practices that work in the US, and reconfigure those that do not, even when those methods have proven to be successful in international MFIs.

As many authors have pointed out, the regulatory framework should be considered because it can contribute to the expansion and enhancement of microfinance in a country (Nayak and da Silva 2019). On the one hand, some scholars reveal that the regulatory framework should be considered because it has positive effects on the microfinance industry (Nayak and da Silva 2019). Additionally, some authors pointed out that the regulatory concerns of the microfinance sector lie in the special nature of these institutions, which caters to the needs of those who have been marginalized by the formal financial sector (Arun 2005).

On the other hand, MFIs with regulated institutions exhibit a lower probability of social failure, whereas fast-growing MFIs appear to show a positive correlation (Dorfleitner et al. 2017). However, as all the analyzed microborrowers in this study develop their activity in the same country (more specifically, in the same state), the legal factor is not differential and, consequently, will not be taken into account.

### Socioeconomic factors

In general, minorities have been another socioeconomic factor analyzed by scholars, especially focusing on the empowerment of women in developing economies and geographic areas such as India, Bangladesh, and some Latin American countries. Specifically, “since the ancient period, women have been under the paws of inequality in our socioeconomic environment” (Dasgupta and Tabassum 2017). In effect, women have been found to suffer more discrimination than other groups, as they have the lowest literacy and employment rates as well as negligible participation in political activities. For a detailed review of the available literature that is relevant to the study of the role of microfinance for women’s empowerment, particularly focused on religious minorities, see Dasgupta and Tabassum (2017).

In developing countries, some scholars (Nannyonga 2000; Papias and Ganesan 2009) claimed that the results from tested empirical models show that age, gender, size of the household, purpose for credit, interest rate charges, and number of official visits to credit societies have a strong effect on loan repayment. On the other hand, the education level of borrowers “should be included to each and every development agenda for the poor since it is key to any positive change and sustainable development of people” (Hadi et al. 2015). This is also highlighted by Bhatt and Tang (2002) and studied in developing countries such as Uganda (Nannyonga 2000).

Research focused on the relationship between microcredits and the education level of borrowers has recently received special attention. Rokhim et al. (2022) pointed out that the lack of education could be due to a variety of factors, including financial instability and fear of abandonment or segregation. These authors concluded that it is impossible to ignore the link between the financial security of families with businesses and their children’s education. It is crucial to examine poverty eradication strategies from the perspective of businesses and parents, as well as to consider the welfare and education of children.

As indicated previously, gender plays a relevant role in microcredits (D’Espallier et al. 2011, 2013; Johnson 2005). Some studies show that women are not only good clients but also better payers than men (Microcreditsummit. Org 2005; Pollinger et al. 2007). Additionally, according to the aforementioned studies, women present higher levels of credit reimbursement than men (Campbell and Rogers 2012). It is therefore worth noting that two-thirds of all MFI borrowers are women and that their businesses exhibit high repayment rates and satisfactory financial results. This justifies the view that this reduced default risk is related to females (Abdullah and Quayes 2016). There is no lack of studies arguing that women outperform men in terms of repayment in microfinance (Bezboruah and Pillai 2017; Bilau and St-Pierre 2018). Some scholars (Agier and Szafarz 2013), who analyzed whether men and women enjoy the same credit conditions by using 34,000 loan applications from a Brazilian microcredit institution, found that there was no gender bias related to loan denial, but that there were different treatments related to credit conditions. Additionally, there are many studies on the relationship between borrowers’ gender and microcredit performance in developing countries (Berg et al. 2015). They conclude that women are more likely to repay collateral-free microloans than men, irrespective of any control mechanisms such as joint liability or dynamic repayment incentives (Shahriar et al. 2020).

Sooryamoorthy (2005) showed that the pattern of credit used by women in the so-called self-help groups is related to their marital status, the difference in this pattern being due to the share of borrowers’ responsibilities with their parents or husbands. The same conclusion was reached by Salazar (2008). However, Santandreu et al. (2020), when analyzing the determinants of repayment among male and female microcredit clients in the USA, showed that based on managers’ perceptions, there are no significant differences in microcredit repayment between women and men due to their marital status. Similarly, Roslan and Mohd Zaini (2009) showed that marital status does not influence the probability of repayment default.

### Financial factors

The purpose of the loan has an undeniable effect on the punctuality of repayments and therefore needs special consideration. Nanayakkara and Stewart (2015) showed that in Sri Lanka, unlike Indonesia, the purpose for which the loan is used is found to be significant when predicting the observance of repayment commitments. Khan and Dewan (2017), using an ordered logit model, hypothesized that the repayment probability of a microloan used for income-generating activities is higher than that of a microloan used for other purposes. The use of borrowed funds by smallholder farmers has been analyzed by Kassegn and Endris (2022), who distinguished between productive and non-productive purposes. Using the Tobit model, these scholars show that the purpose of borrowing has a significant relationship with the loan repayment rate at the 1% significance level. Finally, transportation is an important sector receiving microcredits because one of its main concerns is the reduction of fossil fuels and the use of electric vehicles charged with solar energy to avoid carbon emissions (Kou et al. 2022).

However, the type of loan or credit is another relevant factor, as shown by Newyorkfed.Org (2016). The most widespread microfinance instrument is microcredit or microloan, which consists of the disbursement of small and short-term, non-guaranteed loans to individuals or groups to start or expand their businesses (Khavul 2010). Dorfleitner and Oswald (2016) found evidence that the loan amount and loan term influence the probability of default, with women being better repayers. However, Nartea (2012), using a logistic regression model, showed that the repayment period (more than 1 year or less than 1 year) does not affect microcredit loan repayment. In addition, Mensah (2013) indicated that there is no significant relationship between loan defaults and repayment schedules in MFIs. Finally, using a multinomial logit regression model, Nawai and Shariff (2012) demonstrated that the total loan received significantly affects borrowers’ repayment performance such that higher amounts have a higher probability of being repaid on time.

### Company size

With respect to the size of the companies receiving microcredits, Oke et al. (2007), in an empirical analysis of microcredit repayment in Southwestern Nigeria, showed by using stratified random sampling and linear multiple regression that income affected microcredit repayment. In addition, Nawai and Shariff (2012) found that high total income means a better probability of guaranteeing borrowers’ repayments. However, Roslan and Mohd Zaini (2009) highlighted the insignificant effect of the revenue variable.

Nguta and Huka (2013) demonstrated that among various factors, the number of employees influences the repayment of loans. In the beginning, and referring to our analysis, one can think that this variable is related to revenue. However, Table 7 in “Empirical evidence from the USA microcredit market” section shows that the correlation between both variables is very low (0.1509), which justifies the inclusion of this variable representing company size.

## Microfinance in the USA: some particularities

The United States has been a follower rather than a leader in microfinance (Burrus 2005). Owing to its international success, microfinance has become popular as a means of combatting issues of poverty and economic development. However, despite efforts to establish microfinance programs, the USA has not been as successful as its international counterparts.

In effect, the world of microcredits has been less studied and is much less well known in the USA than in other countries. Existing MFIs in the USA have fallen short of meeting microcredit demand (Rubach et al. 2010). Some scholars argue that, compared to less developed countries, the structure of the USA economy makes the hurdles to starting small businesses much higher, and the microenterprise sector itself is much smaller (Salt 2010). However, the need for microcredits in the USA may never have been greater, since microentrepreneurs have not been served by traditional financial institutions (Rubach et al. 2010).

In the 1980s, the first microcredit loans were granted in the USA, and by the 1990s, MFIs were granted in all 50 states (Richardson 2009). This proliferation may be justified because, in the US market, formal financial institutions have hardly ever accepted borrowers with weak credit histories, insufficient collateral, and little or no business experience. Therefore, alternative vehicles for credit and microcredit programs became necessary and proliferated to serve these markets.

There is no common or formal definition of microenterprises, microcredits, or microloans in the USA. For the Small Business Administration, microcredit is usually a short-term loan of less than $35,000 and a microenterprise is a business with five or less employees (Walker 2011). In 2011, according to the Association for Enterprise Opportunity (AEO) (2013), microbusinesses (as previously defined) represent 92% of all businesses in the USA. Their effect on employment includes a total of 26 million direct workers, which increases to 41.3 million if indirect or induced employment is included. It is estimated that microbusinesses had a total productive economic impact of $5 trillion in 2011, generating $135.5 billion in federal, state, and local government taxes (AEO 2013).

As indicated by Santandreu and López Pascual (2019), the number of microentrepreneurs is uncertain given the lack of a single source of information or census. Burrus (2005) reported that in 1999, in a market study by Accion USA to estimate the number of microbusinesses in the USA, a total of 13.1 million microentrepreneurs were estimated, of which 10.8 million did not receive bank loans for their businesses. This represents an important and unattended market.

Previous research has shown that MFIs exist in all 50 US states and have accumulated extensive experience. However, unlike the Asian and Latin American markets, they are limited in scope and size for an economy such as that of the USA. This research confirms the existence of an important and largely ignored market with a lack of national competition, which makes it attractive due to its size and opportunities for foreign MFIs. However, even when there exists a potential market in need of MFIs services, these institutions face the challenges of fringe (pawn shops, payday lenders, etc.) and traditional banks moving downstream (Walker 2011).

More specifically, microloans and other microfinance products traded in the US market differ from those of other countries: (1) Usually, loans are individual, with very little presence of group loans; and (2) microcredits are characterized by high principals, long terms, and low interest rates with upper limits in most cases.

As concluded by Santandreu et al. (2020), it can also be seen that there is an important difference between global knowledge about the behavior of the reimbursement of microcredits in foreign markets and the knowledge of the behavior of the reimbursement of microloans in the US domestic market and, more specifically, in the reimbursement of microloans granted to women. In effect, unlike other markets and when compared to men, few women are financially served, as they are not the focus of many MFIs. The reflected delinquency in repayment is also somewhat higher than that in other markets. All of this is of utmost importance for the sustainability of MFIs, both for those already in existence and those that want to enter the US market.

From a social point of view, microenterprises have become very important in the creation of wealth in communities that are poorly served by traditional financial services. For example, in 2010, households headed by women, in which at least one person was the owner of a microenterprise, generated between $8,000 and $13,000 more in annual family income than similar households without a business owner (AEO 2013).

From a regulatory point of view and when compared to developing economies, some scholars consider that microfinancing in the USA operates in a largely “grey” area and that there is no solid regulatory body which specifically oversees this activity (Walker 2011). Although microfinancing is largely unregulated in the United States, MFIs must abide by state usury laws, capital-holding requirements, and other banking regulations (Pierce 2013; Raheb 2017). However, in this study, the existence of different regulations does not apply, as all microcredit holders have businesses in the USA.

Salt (2010) conducted another study on the modality of group microcredit in a single study of a major city in the Northeast Pacific region of the USA. This study focuses on women. The findings reveal that, for participating women, the impetus to participate in microcredit does not focus on money, although for women, participation is generally positive as they are offered options, opportunities, and resources previously unavailable.

More recently, Santandreu and López Pascual (2019) presented a novel approach aimed at ascertaining whether MFIs operating in the USA should adapt their microcredit policies based on differences in microcredit reimbursement behavior between women and men. According to managers, the determinants of repayment are similar for men and women, which seems to contradict the conventional findings that women are better repayers.

## Empirical evidence from the USA microcredit market

### Sample

The present analysis is based on a sample composed of 519 microentrepreneurs located in Florida, all of whom were of Hispanic origin (i.e., coming from Hispanic communities in the USA), who obtained microcredits with the aim of covering the financial needs of their respective companies. Respondents were selected through random sampling from January 2018 to December 2019. Specifically, the sample is composed of all microborrowers holding a microcredit granted by the company “OUR Microlending”, which operates in Florida.

### Data

**Fig. 2:**
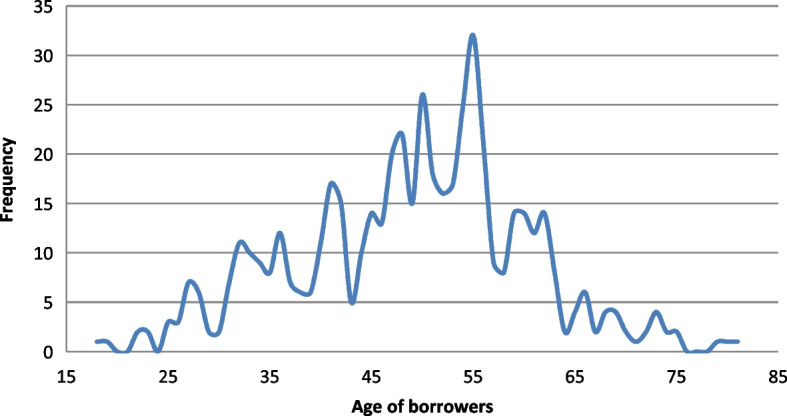
Distribution of the sample by manager ages. *Source*: Own elaboration

**Fig. 3:**
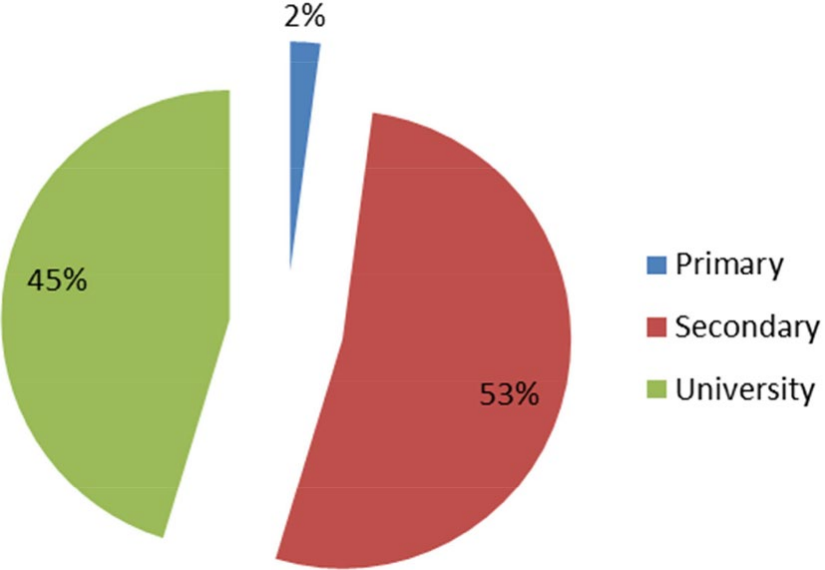
Distribution of the sample by manager education. *Source*: Own elaboration

The data include information on microentrepreneurs, their corresponding companies, and microcredits. First, all entrepreneurs are of Hispanic ethnicity, and the variables considered from their personal information are as follows:

1. Gender ($X_1$): This categorical variable refers to the gender of borrowers and takes the values 1 (for men) and 2 (for women). The sample is composed of 372 men and 147 women.
2. Age ($X_2$): This quantitative variable refers to the age of borrowers (in years) which ranges from 18 to 81 years, 49 years being the approximate average age (see Fig. 2). Observe that the distribution of ages is right skewed, i.e., most borrowers are aged under the mode (55). This means that microloans are more requested by the younger microentrepreneurs in the sample.
3. Education level ($X_3$): This is an ordinal variable which exhibits the following modalities: Primary school (1), Secondary school (2) and University (3) (see Fig. 3). Observe that the education level of microborrowers in the sample is shared equally between Secondary and University.
4. Marital status ($X_4$): Single (1) and married (2). Table 2 shows the marital status of men and women.

**Table 2:** Distribution of the sample by marital status of male and female managers

	Men	Women	Total
Single	177	80	257
Married	195	67	262
Total	372	147	519

*Source*: Own elaboration

**Fig. 4:**
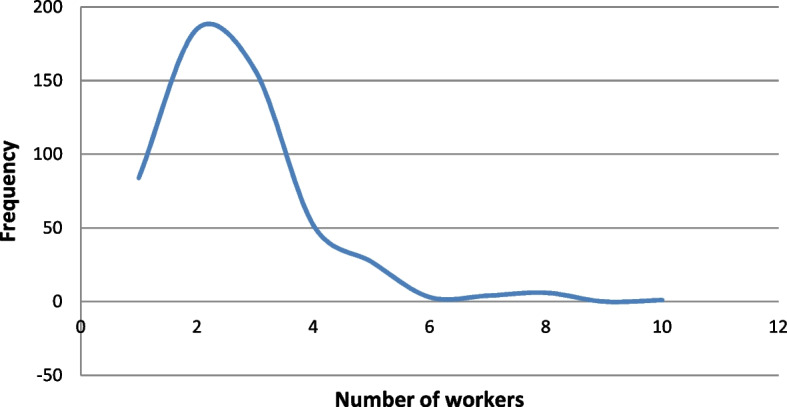
Distribution of companies in the sample by the number of workers. *Source*: Own elaboration

**Fig. 5:**
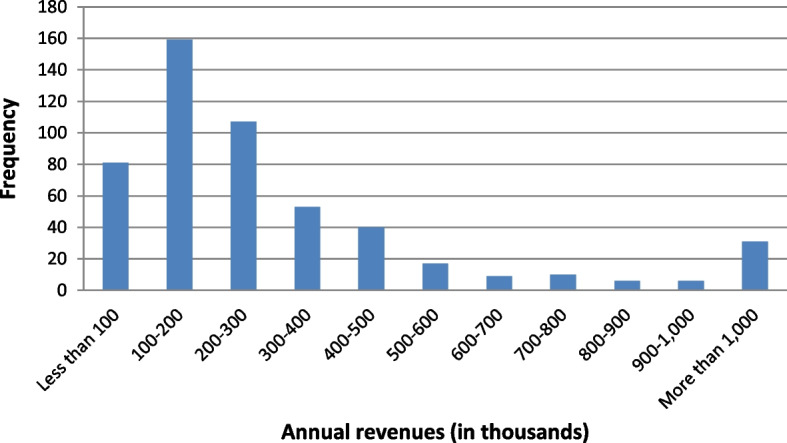
Distribution of companies in the sample by annual revenues. *Source*: Own elaboration

**Fig. 6:**
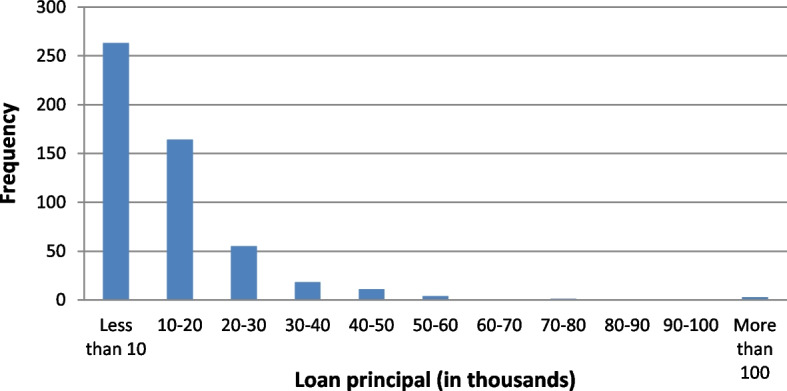
Distribution of the loan principals of companies included in the sample. *Source*: Own elaboration

**Fig. 7:**
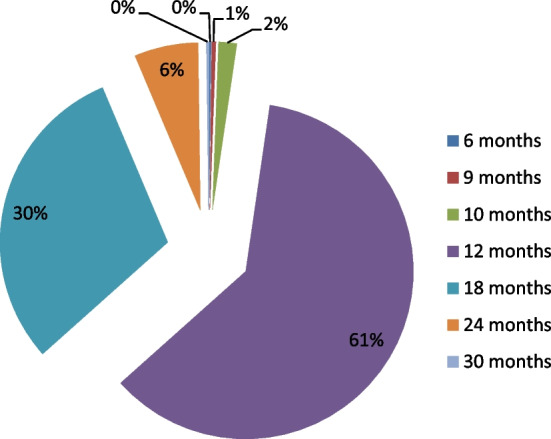
Distribution of the loan terms of companies included in the sample. *Source*: Own elaboration

On the other hand, the variables corresponding to microloans and the general and economic situations of the involved firm are:

1. Closeness to the lender ($X_5$): This ordinal variable refers to the distance between the borrower’s company within Florida and the lender (located in Miami). It is important to highlight that the main locations of borrowers are the counties of Miami-Dade (340 companies), followed by Broward (113) and Orange (5). Borrowers are scored from 1 to 10 according to their proximity to the lender: 1 for the furthest location (Brevard) and 10 for the closest (West Palm Beach). The correspondences are shown in Table 3.
2. Number of workers ($X_6$): This quantitative variable refers to the number of employees. Observe that this variable serves as an indication (proxy) of the economic dimension of the microborrower’s company. This figure ranges from 1 to 10 workers, the average being 2.64 (see Fig. 4). Observe that the distribution of the number of workers is clearly right skewed which means that microloans are largely granted to smaller companies.
3. Revenues ($X_7$): This quantitative variable is classified in intervals from 1 to 11 according to their amounts: 1 for the smallest amount and 11 for the biggest. The assignments are shown in Table 3 and the frequencies of each interval are shown in Fig. 5. The distribution of the annual revenues in the companies in the sample confirms the former statement on Fig. 4.
4. Loan principal ($X_8$): This quantitative variable is classified in intervals from 1 to 11 according to loan principals: 1 for the smallest principal and 11 for the biggest. The assignments are shown in Table 4 and the frequencies of each interval are shown in Fig. 6. All loans are to be repaid by using the French method. The distribution of the loan principals reinforces the idea that the target companies are small and consequently need a smaller amount to develop their businesses.
5. Loan term ($X_9$): The values of this quantitative variable are represented in Fig. 7 (average term: 14.53 months). Observe that most microloans are short (61%) or very short-term (31%).
6. Loan purpose ($X_{10}$): This categorical variable refers to the purpose of loan requested by the borrower’s company. In this way, 430 of them are destined for work capital, 44 for fixed assets, 21 for business improvement, and 24 for a combination of the two.
7. Punctuality of repayments (*Y*): This ordinal variable is divided into good borrowers (434), characterized by on-time repayments, delinquent or in-arrears borrowers (39), and defaulting borrowers (46). This is the dependent variable of the analysis.

**Table 3:** Scores according to revenues

Revenue (in $ US)	Score
Up to $100,000	1
From $100,001 to $200,000	2
From $200,001 to $300,000	3
From $300,001 to $400,000	4
From $400,001 to $500,000	5
From $500,001 to $600,000	6
From $600,001 to $700,000	7
From $700,001 to $800,000	8
From $800,001 to $900,000	9
From $900,001 to $1,000,000	10
From $1,000,001	11

*Source*: Own elaboration

**Table 4:** Scores according to loan principals

Loan principal (in $ US)	Score
Up to $10,000	1
From $10,001 to $20,000	2
From $20,001 to $30,000	3
From $30,001 to $40,000	4
From $40,001 to $50,000	5
From $50,001 to $60,000	6
From $60,001 to $70,000	7
From $70,001 to $80,000	8
From $80,001 to $90,000	9
From $90,001 to $100,000	10
From $100,001	11

*Source*: Own elaboration

Table 5 summarizes the variables (dependent and independent) used in this study by specifying the type of each variable and the potential values they can take. Table 6 shows the expected impact of each variable on loan repayment, supported by the bibliographic references cited in this study.

**Table 5:** Type of variables involved in the analysis

Variable	Short description	Type	Values
*Y*	Punctuality in payments	Ordinal	1, 2, 3
$X_1$	Gender	Categorical	1, 2
$X_2$	Age	Quantitative	18,..., 81
$X_3$	Education level	Ordinal	1, 2, 3
$X_4$	Marital status	Categorical	1, 2
$X_5$	Closeness	Ordinal	1, 2,..., 10
$X_6$	Number of workers	Quantitative	1, 2,..., 10
$X_7$	Revenues	Ordinal	1, 2,..., 11
$X_8$	Loan principal	Ordinal	1, 2,..., 11
$X_9$	Loan term	Quantitative	6, 9, 10, 12, 18, 24, 30
$X_{10}$	Loan purpose	Categorical	1, 2, 3, 4

*Source*: Own elaboration

**Table 6:** Expected impact of each independent variable on the dependent variable

Variable	References	Expected impact on $b_k$
Dependent variable		
Punctuality of repayments		
Independent variables		
Personal information		
Gender	Nannyonga (2000)	
	Kevane and Wydick (2001)	
	Johnson (2005)	
	Pollinger et al. (2007)	
	Papias and Ganesan (2009)	
	Campbell and Rogers (2012)	
	Agier and Szafarz (2013)	
	D’Espallier et al. (2011, 2013)	−
	Ayayi and Sene (2014)	
	Berg et al. (2015)	
	Abdullah and Quayes (2016)	
	Bezboruah and Pillai (2017)	
	Dasgupta and Tabassum (2017)	
	Bilau and St-Pierre (2018)	
	Shahriar et al. (2020)	
Age	Nannyonga (2000)	−
	Papias and Ganesan (2009)	
Education level	Bhatt and Tang (2002)	
	Hadi et al. (2015)	−
	Rokhim et al. (2022)	
Marital status	Sooryamoorthy (2005)	
	Salazar (2008)	−
	Santandreu et al. (2020)	
Firm’s information		
Closeness to the lender	Kassegn and Endris (2022)	−
Number of workers	Nguta and Huka (2013)	$+$
Revenues	Von Pischke (1991)	
	Nannyonga (2000)	
	Oke et al. (2007)	−
	Roslan and Mohd Zaini (2009)	
	Nawai and Shariff (2012)	
Loan’s information		
Loan principal	Dorfleitner and Oswald (2016)	$+$
	Nawai and Shariff (2012)	
Loan term	Dorfleitner and Oswald (2016)	
	Nartea (2012)	$+$
	Mensah (2013)	
Loan purpose	Nanayakkara and Stewart (2015)	
	Khan and Dewan (2017)	−
	Kassegn and Endris (2022)	

*Source*: Own elaboration

A preliminary diagnostic showed that there was no multicollinearity, as all VIF values were smaller than 2.5. The White test shows whether the variance of the errors is constant (homoscedasticity) or not (heteroscedasticity). As the *p* value from this robustness test equals 0.0000, the variance is not homogeneous.

### Methods

The methodology used in this study is the multinomial logit regression method with the aim of analyzing the dependent variable *Y* which identifies three distinct categories. In effect, the dependent variable is divided into three values: $Y = 1$ if there is no problem with repayment, $Y = 2$ if there is delinquency, and $Y = 3$ if there is default. The analysis followed the methodology developed by Agresti and Franklin (2013), Agresti (2015), and Greene (2018).

The criteria for using logistic regression in this study were as follows. First, logistic regression was applied to predict the categorical dependent variable (punctuality of repayments). Second, the predictor variables fall into the following categories: continuous data (revenues and loan principal), discrete nominal data (gender, marital status, and loan purpose), and discrete ordinal data (age, education level, closeness, number of workers, and loan terms) (see Table 1). Third, there was no multicollinearity between the independent variables. The data sample size is large (which allows more accurate results than when the sample is small) and there are no outliers. Freeman (1987) suggested that the sample size should be greater than $10 \times (k + 1)$, where *k* denotes the number of independent variables. In other words, the sample size should be ten times the number of parameters to be estimated, plus one. In our case, $519 > 10 \times (10 + 1)$.

Multinomial logistic regression is an extension of logistic regression for binary outcomes. It starts with *n* independent observations with *p* explanatory variables, where the qualitative response variable has *k* categories. To construct logits in the multinomial case, one of the categories must be considered at the base level, and all logits must be constructed relative to it. Any category can be considered as the base level. Because there is no ordering, category *k* can be considered the base level. Let $\pi _j$ denote the multinomial probability of an observation falling in the *j*th category. The relationship between this probability and the *p* explanatory variables $X_1, X_2, \dots , X_p$ in the multiple logistic regression model is given by

1 $$\begin{aligned} \log \frac{\pi _j(x_i)}{\pi _k(x_i)} = \alpha _{0i} + \beta _{1j} x_{1i} + \beta _{2j} x_{2i} + \cdots + \beta _{pj} x_{pi};\ i = 1, 2, \dots , n;\ j = 1, 2, \dots , k-1, \end{aligned}$$

where, in this case, $j = 1, 2$. Because the sum of all $\pi _j(x_i)$ is one, we have

2 $$\begin{aligned} \pi _j(x_i) = \frac{\exp \{\alpha _{0i} + \beta _{1j} x_{1i} + \beta _{2j} x_{2i} + \cdots + \beta _{pj} x_{pi}\}}{1 + \sum _{h=1}^{k-1} \exp \{\alpha _{0i} + \beta _{1h} x_{1i} + \beta _{2h} x_{2i} + \cdots + \beta _{ph} x_{pi}\}}. \end{aligned}$$

For each $j = 1, 2, \dots , k-1$ (in this case, $j = 1, 2$), the model parameters were estimated using the maximum likelihood method. If $\mathbf {x}$ denotes a matrix or vector, let $\pi _j(\mathbf {x}) = P(Y = j / \mathbf {x})$ for a given setting $\mathbf {x}$ of the explanatory variables, where $\sum _{j=1}^{k-1} \pi _j(\mathbf {x}) = 1$. The logit models pair each response category with a baseline category.

3 $$\begin{aligned} \log \frac{\pi _j(\mathbf {x})}{\pi _k(\mathbf {x})} = \alpha _j + \varvec{\beta }_j^{\prime } \mathbf {x}, \end{aligned}$$

where $j = 1, 2, \dots , k-1$ (in this case, $j = 1, 2$) simultaneously describes the effects of $\mathbf {x}$ on these $k-1$ logits. As the effects vary according to the response paired with the baseline, these $k-1$ equations determine the parameters for the logits with other pairs of response categories. Finally, the Pearson chi-square statistic $\chi ^2$ and the likelihood ratio chi-square statistic $G^2$ goodness-of-fit statistics provide a model check when data are not scarce.

Another methodological possibility in credit evaluation is to find clusters that help to adequately interpret the available financial data to identify users’ behaviors and potential risks (Li et al. 2021). Similarly, Wang et al. (2021) introduced a credit rating for peer-to-peer (P2P) lending using a multi-class classification model. This is very important because, as in our study, standard binary classifiers are not appropriate for controlling default risk in P2P lending, which leads to misclassification costs.

### Results

The following null and alternative hypotheses are tested by applying the methodology described in “Methods” section:

- $$H_0: \log (\text{ odds}) = b_0$$
- $H_1: \log (\text{ odds}) = b_0 + b_1 X_1 + \cdots + b_p X_p$, where some $b_j$ are different from zero.

As indicated, the proposed model aims to maximize log-likelihood. Thus, the log-likelihood for the model is $LL_1 = -271.9468$, whereas that for the model with only the constant terms is $LL_0 = -290.0404$. In other words, the likelihood ratio statistic for the hypothesis that all 10 coefficients of the model are zero is greater than the critical value. The outputs of the model are listed in Table 7.

**Table 7:** Coefficients relating category 3 to category 1

Coefficient	Value	S.E.	z-stat	Lower $z_{0.025}$	Upper $z_{0.975}$	$\exp \{b_k\}$	*p* Value
$b_0$	− 1.8217	1.9418	− 0.9381	$-5.6276$	1.9843	0.1618	0.3482
$b_1$	0.3832	0.3433	1.1164	$-0.2896$	1.0561	1.4670	0.2643
$b_2$	− 0.0317	0.0150	− 2.1191	$-0.0610$	− 0.0024	0.9688	0.03409 (**)
$b_3$	0.2365	0.3016	0.7844	$-0.3545$	0.8276	1.2669	0.4328
$b_4$	− 0.5569	0.3372	− 1.6518	$-1.2177$	0.1039	0.5730	0.0986 (*)
$b_5$	0.1254	0.1664	0.7537	$-0.2008$	0.4516	1.1336	0.4511
$b_6$	− 0.0762	0.1360	− 0.5605	$-0.3428$	0.1903	0.9266	0.5751
$b_7$	0.0335	0.0719	0.4664	$-0.1073$	0.1743	1.0341	0.6409
$b_8$	− 0.2520	0.2148	− 1.1732	$-0.6729$	0.1690	0.7773	0.2407
$b_9$	0.0455	0.0505	0.9017	$-0.0534$	0.1444	1.0466	0.3672
$b_{10}$	− 0.4302	0.2801	− 1.5359	$-0.9791$	0.1188	0.6504	0.1246

*Source*: Own elaboration

*Significant at 10% level, **significant at 5% level. *S.E.* standard error

The information in Table 7 can be interpreted as follows:

- One unit increase in $X_2$ will decrease the odds of 3 in comparison to 1 by 3.1% (i.e., the odds will be multiplied by 0.9688).

**Table 8:** Coefficients relating category 2 to category 1

Coefficient	Value	S.E.	z-stat	Lower $z_{0.025}$	Upper $z_{0.975}$	$\exp \{b_k\}$	*p* Value
$b_0$	− 1.9121	1.5389	− 1.2425	− 4.9282	1.1040	0.1478	0.2140
$b_1$	− 0.2890	0.4289	− 0.6739	− 1.1295	0.5516	0.7490	0.5004
$b_2$	− 0.0151	0.0154	− 0.9794	− 0.0454	0.0151	0.9850	0.3274
$b_3$	− 0.4577	0.3385	− 1.3520	− 1.1212	0.2058	0.6328	0.1764
$b_4$	0.2180	0.3550	0.6140	− 0.4779	0.9138	1.2436	0.5392
$b_5$	− 0.1528	0.1112	− 1.3739	− 0.3708	0.0652	0.8583	0.1695
$b_6$	0.1876	0.1185	1.500	− 0.0448	0.4199	1.2063	0.1136
$b_7$	− 0.0115	0.0811	− 0.1421	− 0.1705	0.1474	0.9885	0.8870
$b_8$	− 0.0981	0.1974	− 0.4972	− 0.4850	0.2887	0.9065	0.6190
$b_9$	0.1717	0.0469	3.6590	0.0797	0.2636	1.1873	0.0003 (***)
$b_{10}$	− 0.1374	0.2604	− 0.5277	− 0.6478	0.3730	0.8716	0.5977

*Source*: Own elaboration

***significant at 1% level. *S.E.* standard error

Similarly, the information in Table 8 can be interpreted as follows:

- One unit increase in $X_9$ will increase the odds of 2 in comparison to 1 by 18.73% (i.e., the odds will be multiplied by 1.1873).

Taking into account that the scores assigned to the dependent variable increase with the delay in loan repayment, a negative coefficient indicates that the corresponding variable is associated with a probability of being in the category “on-time repayment” greater than being in the category of “delinquent or defaulted borrowers”. Conversely, a positive coefficient indicates that the involved variable is associated with a probability of being in the category of “good borrowers” lower than being in the category of “bad borrowers”.

The results of this study have both theoretical and practical implications for the Hispanic Latin minority because they clearly show that a one-unit increase in age, education level, marital status, company proximity, loan principal, and loan purpose implies a higher probability of being “good borrowers” than “bad borrowers”. In contrast, a one-unit increase in gender, company location, number of workers, revenues, and loan term purpose implies a lower probability of being “good borrowers” than “bad borrowers”. Most of the coefficients, except the age and loan term, are not statistically significant, but the results obtained in this sample clearly indicate the direction to be followed in further research on this topic.

The symmetric matrix in Table 9 reflects the correlation between the numerical, ordinal, and dichotomous (nominal) predictors used in the model.

- The Spearman rank order correlation coefficient between numerical, ordinal or dichotomous (nominal) predictors, and ordinal or dichotomous (nominal) ones.
- The Pearson correlation coefficient between numerical predictors.

The variable $X_{10}$ has not been included in Table 9 because it is a non-dichotomous (nominal) predictor; therefore, its association with other variables requires the calculation of the corrected coefficient of contingency, whose nature is different from the other two correlation coefficients.

**Table 9:** Matrix of correlations between the independent variables

$X_k$	$X_1$	$X_2$	$X_3$	$X_4$	$X_5$	$X_6$	$X_7$	$X_8$	$X_9$
$X_1$	1.0000								
$$X_2$$	0.0879$$^{**}$$	1.0000							
$X_3$	0.0208$^{*}$	0.0826$^{*}$	1.0000						
$$X_4$$	$$-0.0617$$	0.1739$$^{***}$$	0.0093	1.0000					
$$X_5$$	0.1240$$^{***}$$	0.0314	$$-0.0245$$	0.0090	1.0000				
$$X_6$$	0.1200$$^{***}$$	0.0996$$^{***}$$	0.1769$$^{***}$$	$$-0.0385$$	$$-0.0211$$	1.0000			
$$X_7$$	$$-0.1070$$ $$^{**}$$	$$-0.0537$$	0.1070$$^{**}$$	0.0952$$^{**}$$	$$-0.0464$$	0.1649$$^{***}$$	1.0000		
$$X_8$$	$$-0.1275$$ $$^{***}$$	$$-0.0550$$	0.1149$$^{***}$$	0.104$$^{**}$$	$$-0.1096$$ $$^{**}$$	0.0235	0.4937$$^{***}$$	1.0000	
$$X_9$$	$$-0.06677$$	0.0769$$^{*}$$	0.0017	0.0274	$$-0.0669$$	0.0250	0.1717$$^{***}$$	0.4853$$^{***}$$	1.0000

*Source*: Own elaboration

$$^{*}$$Significant at 10% level, $$^{**}$$significant at 5% level, $$^{***}$$significant at 1% level

In the estimated parameters, the independent $X_1$, $X_3$, $X_4$, $X_5$, $X_6$, $X_7$, $X_8$ and $X_{10}$ variables are not significant predictors for *Y*. However, it appears that only age and loan term are statistically significant. This indicates that marketing experts should focus on these two variables to increase their market share.

With respect to the relationship between odds and independent variables, we have:

- McFadden $$R^2$$ equals 0.06238.
- Cox and Snell $R^2$ equals 0.06735.
- Nagelkerke $$R^2$$ equals 0.06735.

However, the scarcity of information on the three former statistical measures suggests the following. Regarding the goodness of fit of the overall regression, the right tail is given by $\chi ^2(20) = 36.1872$ and *p* value = 0.01462. Since *p* value $< 0.05$, $H_0$ can be rejected. Therefore, the impact of some coefficients of the logistic regression model $\log (\text{ odds}) = b_0 + b_1 X_1 + \cdots + b_p X_p$ is statistically different from zero.

With respect to the factors affecting the quality of repayment of microcredits in Florida State (United States), the results show, after applying the stated methodology, that age positively affects the repayment of microloans in the selected sample by exhibiting a negative coefficient, while loan term exhibits a positive coefficient, as shown in Tables 7 and 8, and consequently have a negative effect.

## Discussion

In this study, multinomial logit (MNL) regression was employed as a suitable methodology when the dependent variable was categorical and presented more than two possible discrete results. Additionally, this method permits the calculation of the log odds of the outcomes as a linear combination of several predictor variables, taking real, binary, or categorical values. This methodology has been extensively used in the literature on this topic (for example Nartea 2012; Nawai and Shariff 2012; Dorfleitner and Oswald 2016; Khan and Dewan 2017; Kassegn and Endris 2022).

This is a very particular context that may differ from other studies on this topic, which have focused on similar factors but in developing countries such as Malaysia, India, and Tunisia (Nawai and Shariff 2012; Sangwan et al. 2020; Baklouti 2013). The results of the study carried out in Malaysia show that there are ten factors affecting borrowers’ repayment performance. In effect, age, formal religious education, total income, and business formality have negative coefficients, while gender, business experience, distance and frequency of visits to the lender office, and loan approval have positive coefficients. Other scholars (Baklouti 2013) concluded that borrowers’ socio-demographic characteristics, previous participation in microcredit loans, and past credit history have significant effects on their default rates.

Theoretically, these factors indicate some socioeconomic differences between the Hispanic minority and other generally perceived Western business cultures (Zhang and López Pascual 2012), and underline the importance of distinguishing the main factors to be considered when identifying microcredit policies.

The results also confirm the significance of the borrowers’ profile in that the older the borrowers, the more responsible they are in repaying the loan. This finding supports the idea that age is a measure of experience, and this helps identify the appropriate principal and term of the corresponding loan. Additionally, the study shows that the loan repayment period is significant at the 1% level in such a way that the loan term increases the probability of borrowers’ delinquency. Finally, as indicated in Table 7, marital status is significant at the 10% level, which means that married borrowers have a greater probability of repaying on time instead of falling into default. This coincides with the results expected from the literature on the topic (see Table 6).

However, it has not been demonstrated that the distance between the lender and borrower affects loan repayments (Bhatt and Tang 2002; Oke et al. 2007; Derban et al. 2005). With respect to revenue, there is no direct relationship between this variable and the probability of punctuality of repayments, which is contrary to the conclusions of Von Pischke (1991) and Nannyonga (2000). Additionally, as expected, the loan principal is not directly related to the probability that borrowers repay their loans on time. In particular, regarding gender, the female borrowers do not exhibit a higher probability of being in the delinquent category.

## Conclusions, recommendations, policy implications and future research

Microcredits have shown to be an essential source of funding in many developing countries in Asia (Ali et al. 2020) and Latin America. However, nothing has been said about the profiles of microcredit holders in these countries. Indeed, this is an important issue to be considered to indicate the probability of success or failure of these financing sources. However, the main problem in identifying the profile of microcredit holders in these countries is the scarcity of information on the characteristics of these microentrepreneurs and their corresponding companies.

A possible solution is to analyze the main demographics in order to obtain some characteristics of entrepreneurs coming from Hispanic communities in other developed markets where there is easy access to information. This justifies the main objective of the present study, which is to identify the main characteristics of Latin microentrepreneurs, all of them of Hispanic origin (i.e., coming from Hispanic communities in the USA), and even the main characteristics of their companies, particularly to consider a series of factors that will help explain the repayment characteristics of their respective microloans. Indeed, this will be an initial step before drawing conclusions to apply to developing countries rather than developed countries.

Given the interest in exploring the main socioeconomic factors affecting the quality of repayment of microcredits in developed economies, this study aims to identify how these factors influence the profile of microcredit holders, especially in developing markets in the USA. In effect, this study highlights the relevance of the main factors that affect the repayment of microcredits to achieve better performance by employing a multinomial logit regression. Owing to the nature of the methodology employed, the statistical generalization of the results needs to be considered with caution, especially in terms of socioeconomic factors. In effect, there are different approaches to analyzing the profile of microcredit clients, specifically those of Hispanic ethnicity, which point out the importance of distinguishing the main factors to achieve better results in microcredit policies.

This demonstrates that age is linked to more responsible loan repayment. Additionally, the results support the view that the loan term is inversely related to the probability that borrowers will repay their loans on time.

It also aims to offer a dynamic socioeconomic approach to the profile of microcredit holders of the Hispanic minority in the USA to assist lenders in identifying more reliable borrowers by employing the methodology of multinomial logit regression.

From the perspective of practitioners, this research offers a practical guide for MFIs oriented towards the Hispanic minority by identifying the most appropriate profiles that lead to on-time repayment of loans.

There is no doubt that the rapid growth of the Hispanic population in the United States will result in increased political influence and representation. The recent Biden Administration has pointed out that “the Latino community is a core part of the American story and their contributions are evident in every part of society” (see https://joebiden.com/todos-con-biden-policy/). Latin Americans also created new enterprises, owning nearly one in four new businesses (Mills et al. 2018). In addition to promoting economic growth, Latin Americans are major tax contributors, funding over $215 billion in tax revenues, including $76 billion in state and local taxes (New American Economy 2017). Despite this economic progress, Latin Americans continue to face significant economic challenges as they are 1.7 times more likely than whites to live in poverty (Maloney 2019).

However, the results of this study may have a limitation in their application to other contexts because the research was limited to Florida. With this limitation, future research is desirable and feasible. Some useful future research could analyze the profile of Hispanic minority microcredit holders and companies in other states with a higher Hispanic population, such as New Mexico, Texas, California, Arizona, Nevada, and Colorado, and thereby determine the main factors that influence the repayment behavior among male and female microcredit borrowers in this minority group in the USA.

It is hoped that this study will contribute to a better general understanding of these socioeconomic factors. The conclusion of this study revisits the literature on the factors affecting the repayment of microcredits for some specific minorities in the sample studied.

Some potential future research could be, but is not limited to, specifically studying, from a socioeconomic point of view, the profile of Hispanic minority microcredit holders and companies in some other states, besides Florida, with the highest Hispanic population. This is the case for the top states displaying the largest Hispanic populations, such as New Mexico, Texas, California, Arizona, Georgia, Colorado, Illinois, New Jersey, and New York (according to the Census Bureau 2022 and the US Department of Health and Human Services. Office of Minority Health 2022), where it would be interesting to determine the main factors that influence repayment behavior among male and female microcredit borrowers for this minority in the USA. Another possible further research could be to combine the socioeconomic factors displayed in this manuscript with the bankruptcy prediction model for small and medium-sized enterprises (SMEs) introduced by Kou et al. (2021), as it uses transactional data and payment network-based variables under a scenario where no financial data are required.

## Data Availability

The datasets used and/or analysed during the current study are available from the corresponding author on reasonable request.

## Abbreviations

AEO: Association for Enterprise Opportunity
EESG: Economic, environmental, social, and governance
MFI: Microfinance institution
MNL: Multinomial logit
SMEs: Small and medium-sized enterprises
US: United States
USA: United States of America
